# Molecular Characterization and Antimicrobial Resistance in *Neisseria gonorrhoeae*, Nunavut Region of Inuit Nunangat, Canada, 2018–2019

**DOI:** 10.3201/eid2706.204407

**Published:** 2021-06

**Authors:** Ameeta E. Singh, Jasmine Pawa, Kethika Kulleperuma, Errol Prasad, Sonia Marchand, K. Dionne, Maxim Trubnikov, Tom Wong, Michael R. Mulvey, Irene Martin

**Affiliations:** University of Alberta, Edmonton, Alberta, Canada. (A.E. Singh);; Government of Nunavut, Iqaluit, Nunavut, Canada (J. Pawa, K. Kulleperuma);; DynaLIFE Medical Labs, Edmonton (E. Prasad);; Qikiqtani General Hospital Laboratory, Iqaluit, Nunavut (S. Marchand, K. Dionne);; Indigenous Services Canada, Ottawa, Ontario, Canada (M. Trubnikov, T. Wong);; National Microbiology Laboratory, Winnipeg, Manitoba, Canada (M.R. Mulvey, I. Martin)

**Keywords:** antimicrobial resistance, Arctic, bacteria, Canada, DNA, gonorrhea, Inuit Nunangat, molecular characterization, *Neisseria gonorrhoeae*, sequence analysis, sexually transmitted infections

## Abstract

We assessed antimicrobial resistance (AMR) in *Neisseria gonorrhoeae* in Nunavut, Canada, using remnant gonorrhea nucleic acid amplification test–positive urine specimens. This study confirms the feasibility of conducting *N. gonorrhoeae* AMR surveillance and highlights the diversity of gonococcal sequence types and geographic variation of AMR patterns in the territory.

In 2015, the prevalence of *Neisseria gonorrhoeae* reported in the territory of Nunavut, Canada, 837.6 cases/100,000 residents, was 15 times the national rate in Canada (55.4 cases/100,000 residents) (*1*). Gonorrhea is a notifiable disease in Nunavut, and all positive cases are reported to the department of health. Globally, reports are increasing of *N. gonorrhoeae* with resistance to currently recommended first-line antimicrobial treatment agents (*2*). These resistance rates in some global regions approach or exceed World Health Organization thresholds of 5% to warrant changes to the prescribed gonorrhea treatments (*3*).

The Gonococcal Antimicrobial Surveillance Program of Canada is a culture-based laboratory surveillance system monitoring antimicrobial resistance (AMR) trends and gonococcal sequence types (STs) in *N. gonorrhoeae* (*4*). Traditionally, *N. gonorrhoeae* AMR surveillance has required obtaining cultures from patients for phenotypic testing. In the Canadian Arctic regions, including Nunavut, no information has been available on the prevalence and distribution of *N. gonorrhoeae* AMR patterns because long transport times and conditions preclude transporting cultures; assessment is exclusively done by using nucleic acid amplification tests (NAATs). The National Microbiology Laboratory (Winnipeg, Manitoba, Canada) has addressed this issue by developing molecular assays to predict AMR and molecular STs from remnant NAAT specimens. We sought to assess the prevalence and distribution of AMR *N. gonorrhoeae* STs in Nunavut using NAAT-tested *N. gonorrhoeae–*positive specimens. 

## The Study

Nunavut, at 1,877,787 km^2^ the largest territory in Canada, is divided into 3 regions: Qikiqtaaluk (also called Qikiqtani) in the east, Kivalliq in the center, and Kitikmeot in the west. Nunavut’s reported 2016 population was 35,994 (*5*), ≈85% of whom identify as Inuit (*6*). Nunavut is one region in Inuit Nunangat, a Canadian Inuktitut term inclusive of the land, water, and ice of the Inuit homeland (*7*).

For this study we used remnant *N. gonorrhoeae* NAAT–positive specimens collected in Nunavut for routine gonococcal diagnostics during January 1, 2018–December 31, 2019. We submitted specimens from the Kitikmeot region to DynaLife Laboratories (https://www.dynalife.ca) and from the Qikiqtaaluk and Kivalliq regions to the Qikiqtani General Hospital Laboratory (Iqaluit, Nunavut, Canada) to the National Microbiology Laboratory for molecular antimicrobial susceptibility prediction using NAATs and *N. gonorrhoeae* multiantigen sequence typing (NG-MAST). Urine specimens submitted to DynaLife laboratories were tested using the Gen-Probe Aptima Combo 2 test (Hologic, https://www.hologic.com). Testing at the Qikiqtani General Hospital Laboratory was done using the Roche-Cobas test (Roche Molecular Diagnostics, https://diagnostics.roche.com). We did not submit repeat samples or test-of-cure specimens. Ethics approval was obtained from the University of Alberta’s Health Research Ethics Board and the Nunavut Research Institute. 

We performed NG-MAST as described elsewhere (*8*,*9*). We then tested specimens successfully typed with NG-MAST using single-nucleotide polymorphism (SNP) assays targeting cephalosporin-DS mutations (*ponA*, *mtrR* delA, *porB*, *penA* A311V, *penA* A501, *penA* N513Y, and *penA* G545S), ciprofloxacin-resistance mutations (*gyrA* and *parC*), and azithromycin-resistance mutations (23S rRNA A2059G, C2611T, and *mtrR*) to predict antimicrobial susceptibility. We performed DNA extraction, preparation, real-time PCR, and results analysis as described elsewhere (*9*,*10*). For specimens identified with the same ST, we performed SNP assay testing for a subset of samples and for the remainder of samples within identical ST groups. We inferred AMR profiles based on STs with 4–70 samples if ≥50% of those samples had identical AMR predictions based on SNP results and STs with >70 samples if ≥30 samples were tested with identical AMR predictions. These results are molecular based and have been validated against MIC phenotypes in a previous study (*9*).

**Figure 1:**
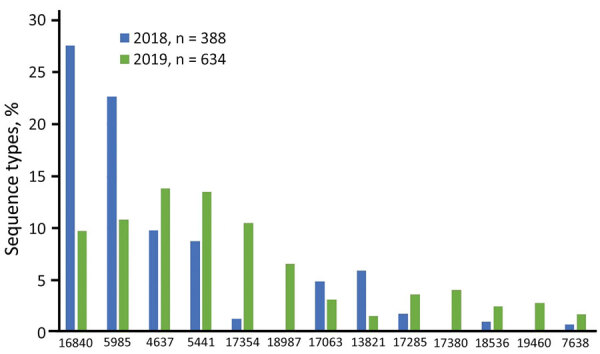
Distribution of prevalent *Neisseria* *gonorrhoeae* multiantigen sequence typing sequence types of gonorrhea-positive nucleic acid amplification specimens (n = 1,022) from a study of antimicrobial resistance in *N. gonorrhoeae* in the Nunavut region of Inuit Nunangat, Canada, 2018–2019.

All NAAT-tested *N. gonorrhoeae*–positive specimens (n = 1,128) from Nunavut collected between January 1, 2018–December 31, 2019, were included in the study (Table 1). Of these, 106 (9.4%) samples were nontypeable for NG-MAST because of low concentrations of DNA and therefore excluded from further testing. We identified a total of 75 different STs among the remaining 1,022 NAAT-identified isolates from samples submitted from Nunavut (Figure 1). The most prevalent was ST16840 (16.5%), followed by ST5985 (15.3%). 

**Table 1:** Sex and geographic distribution of gonorrhea nucleic acid amplification test positive specimens from Nunavut, Canada, 2018–2019*

Year		Region			Total by sex	Total
	Sex	Kivalliq	Qikiqtaaluk	Kitikmeot		
2018	M	62	112	8	182	432
	F	98	135	17	250	
2019	M	94	166	14	274	696
	F	142	256	22	420	
	Unk	0	0	2	2	
Total		396	669	63	1,128	
*Unk, unknown						

**Figure 2:**
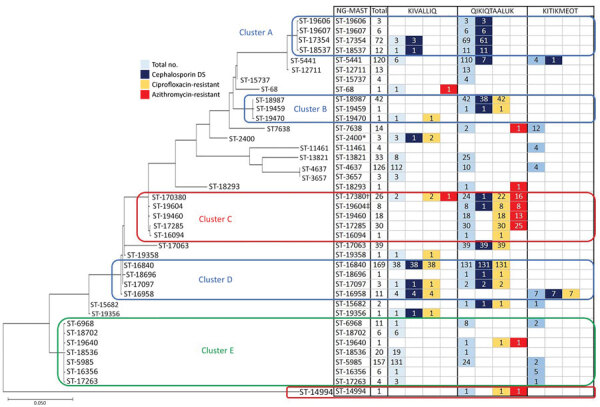
Genetic relationship of *Neisseria* *gonorrhoeae* multiantigen sequence typing sequence types (STs) of gonorrhea-positive nucleic acid amplification specimens with prevalent and predicted nonsusceptible SNP assay results (n = 975) from a study of antimicrobial resistance in *N. gonorrhoeae* in the Nunavut region of Inuit Nunangat, Canada, 2018–2019. Only prevalent STs or STs whose samples predicted decreased susceptibility to cephalosporins or resistance to ciprofloxacin or azithromycin were included. The evolutionary history was inferred by using the maximum-likelihood method and Tamura-Nei model (*13*). The tree with the highest log likelihood (–4321.41) is shown. Initial trees for the heuristic search were obtained automatically by applying neighbor-joining and BioNJ algorithms to a matrix of pairwise distances estimated using the maximum composite likelihood approach, and then selecting the topology with superior log likelihood value. The tree is drawn to scale, with branch lengths measured in the number of substitutions per site. This analysis involved 39 nucleotide sequences. Codon positions included were 1st+2nd+3rd+Noncoding; a total of 917 positions were in the final dataset. Evolutionary analyses were conducted in MEGA X (*14*). Clusters were identified as STs with ≤5 base pair differences between them. *One of the ST2400 samples was predicted to be cephalosporin decreased susceptibility (not cephalosporin/DS). †The sample that was predicted to be cephalosporin/DS was not azithromycin resistant. ‡The sample that was cephalosporin/DS was also azithromycin resistant. Cephalosporin/DS, cephalosporin intermediate/decreased susceptibility.

SNP assay results for predicting AMR were successfully determined for cephalosporin (687 samples), ciprofloxacin (541 samples), and azithromycin (435 samples). We predicted AMR for an additional 289 cephalosporin results, 436 ciprofloxacin results, and 533 azithromycin results on the basis of expected results of prevalent STs tested (Table 2). Within Nunavut, the Qikiqtaaluk region had the highest prevalence of intermediate cephalosporin MICs (51.3%) and resistance to azithromycin (11.3%). The Kivalliq region had the only sample (0.3%) with predicted decreased susceptibility to cephalosporins. We inferred the genetic relationships between the NG-MAST STs by using the maximum-likelihood method and Tamura-Nei model (Figure 2) (*11*,*12*). We predicted most samples in clusters A, B, and D would have elevated MICs to cephalosporins but that most samples in cluster C would have azithromycin resistance. We also found that STs were clustered based on geography: in Qikiqtaaluk, we primarily identified STs from clusters A, B and C, but we identified STs from cluster D in all 3 jurisdictions. 

**Table 2:** Predicted AMR of gonorrhea positive nucleic acid amplification specimens from Nunavut, 2018–2019*

Resistance	MICs, mg/L	No. specimens with AMR/no. predicted (%)			
		Qikiqtaaluk	Kivalliq	Kitikmeot	Total
Decreased susceptibility to cephalosporins	≥0.125	0/591 (0)	1/350 (0.3)	0/35 (0)	1/976 (0.1)
Intermediate cephalosporin MICs	0.032–0.063	303/591 (51.3)	49/350 (14.0)	8/35 (22.9)	360/976 (36.9)
Ciprofloxacin resistant	≥1	298/591 (50.4)	50/351 (14.2)	7/35 (20.0)	355/977 (36.3)
Azithromycin resistant	≥2	66/583 (11.3)	2/350 (0.6)	0/35 (0)	68/968 (7.0)
*Denominators indicate predicted number of samples with susceptibility for the given antimicrobial. Not all samples were tested for antimicrobial susceptibilities and not all the samples that were tested gave conclusive results. AMR, antimicrobial resistance.					

Our analysis of routinely collected diagnostic specimens for gonorrhea from Nunavut highlights the usefulness of SNP assays and NG-MAST typing in identifying population-level *N. gonorrhoeae* AMR and delineating transmission patterns (*9*). Current Nunavut treatment guidelines for gonorrhea recommend dual ceftriaxone/azithromycin therapy (*13*). Although decreased susceptibility to cephalosporins was identified in only 1 sample, the rate of predicted intermediate cephalosporin MICs in Nunavut at 36.9% is >2 times as high as the national rate of 16% in 2018, potentially limiting the long-term use of this agent (*4*). In addition, the 7.0% rate of predicted azithromycin resistance exceeds the World Health Organization threshold of 5% above which a drug is not routinely recommended for treatment (*3*). We also noted substantial geographic variation; the highest prevalence of predicted intermediate cephalosporin MICs (51.3%) and predicted resistance to azithromycin (11.3%) were in the Qikiqtaaluk region. In the adjacent province of Quebec, the reported rate of intermediate cephalosporin MICs was 22% and azithromycin resistance was 11.8% (*4*). NG-MAST typing detected a large number of STs, reflecting the genetic diversity of *N. gonorrhoeae* and suggesting the introduction of multiple strains into the territory. 

The first limitation of our study was that no gonococcal cultures were available and cases were compared to national gonococcal cultures, which may not be representative of gonorrhea in all regions. In addition, only urine specimens were collected for gonorrhea in Nunavut. Previous research has highlighted the variability in ST from extragenital sites (*14*,*15*). There are limitations to using culture-independent techniques to predict AMR because specimens often contain low concentrations of DNA, which can limit SNP detection. Gonococcus is also known to mutate easily, which may lead to false-negative results in the SNP assays because of sequence variations in DNA (*9*). A molecular-based approach may overestimate AMR because mutations associated with resistance do not always correlate phenotypically. Finally, we acknowledge the biomedical and technical laboratory approach of this work and the limited partnerships with the Inuit people. 

## Conclusions 

Because research across Inuit Nunangat has historically often been done in an exploitative way that has not respected Inuit self-determination, meaningful partnerships are required to prioritize research activities and develop approaches that add value for and are accepted by communities. Our findings highlight the feasibility of conducting molecular surveillance for *N. gonorrhoeae* AMR using remnant *N. gonorrhoeae*–positive NAAT specimens Data based on these findings can be used to provide Nunavut with up-to-date information about the best choices to treat gonorrhea. 
